# Physiologically based pharmacokinetic modelling and simulation to predict the plasma concentration profile of schaftoside after oral administration of total flavonoids of *Desmodium styracifolium*

**DOI:** 10.3389/fphar.2022.1073535

**Published:** 2022-12-14

**Authors:** Xue Li, Chao Chen, Nan Ding, Tianjiao Zhang, Peiyong Zheng, Ming Yang

**Affiliations:** ^1^ Phase I Clinical Research Lab, LongHua Hospital, Shanghai University of Traditional Chinese Medicine, Shanghai, China; ^2^ Clinical Research Center, LongHua Hospital, Shanghai University of Traditional Chinese Medicine, Shanghai, China

**Keywords:** physiologically based pharmacokinetic model, schaftoside, total flavonoids of *Desmodium* styracifolium, urolithiasis, rat, human

## Abstract

**Introduction:** The total flavonoids of *Desmodium styracifolium* (TFDS) are the flavonoid extracts purified from Desmodii Styracifolii Herba. The capsule of TFDS was approved for the treatment of urolithiasis by NMPA in 2022. Schaftoside is the representative compound of TFDS that possesses antilithic and antioxidant effects. The aim of this study was to develop a physiologically based pharmacokinetic (PBPK) model of schaftoside to simulate its plasma concentration profile in rat and human after oral administration of the total flavonoids of *Desmodium styracifolium*.

**Methods:** The physiologically based pharmacokinetic model of schaftoside was firstly developed and verified by the pharmacokinetic data in rats following intravenous injection and oral administration of the total flavonoids of *Desmodium styracifolium*. Then the PBPK model was extrapolated to human with PK-Sim^®^ software. In order to assess the accuracy of the extrapolation, a preliminary multiple-dose clinical study was performed in four healthy volunteers aged 18–45 years old. The predictive performance of PBPK model was mainly evaluated by visual predictive checks and fold error of C_max_ and AUC_0-t_ of schaftoside (the ratio of predicted to observed). Finally, the adult PBPK model was scaled to several subpopulations including elderly and renally impaired patients.

**Results:** Schaftoside underwent poor metabolism in rat and human liver microsomes *in vitro*, and *in vivo* it was extensively excreted into urine and bile as an unchanged form. By utilizing literature and experimental data, the PBPK model of schaftoside was well established in rat and human. The predicted plasma concentration profiles of schaftoside were consistent with the corresponding observed data, and the fold error values were within the 2-fold acceptance criterion. No significant pharmacokinetic differences were observed after extrapolation from adult (18–40 years old) to elderly populations (71–80 years) in PK-Sim^®^. However, the plasma concentration of schaftoside was predicted to be much higher in renally impaired patients. The maximum steady-state plasma concentrations in patients with chronic kidney disease stage 3, 4 and 5 were 3.41, 12.32 and 23.77 times higher, respectively, than those in healthy people.

**Conclusion:** The established PBPK model of schaftoside provided useful insight for dose selection of the total flavonoids of *Desmodium styracifolium* in different populations. This study provided a feasible way for the assessment of efficacy and safety of herbal medicines.

## 1 Introduction

Urolithiasis, also known as urinary calculi or stones, is a widespread disease. It is the most frequent diagnosis in the urology departments, and affects 1–20% of the adult population (Li et al., 2020). The prevalence rate of urolithiasis is approximately 5–9% in Europe, 12–15% in North America, and 1–5% in Asia (Ramello et al., 2000; López and Hoppe, 2010). Moreover, the relapse rate of this disease is approximately 50% within the subsequent 5–10 years after the first episode (Fisang et al., 2015). Surgical managements are widely applied to remove the calculi in patients (Jiang et al., 2021). However, the risk of a symptomatic stone episode remains an important unresolved problem due to the stone fragments remaining in the urinary system after the intervention (Ozdedeli and Cek, 2012). To facilitate the passage of ureteral stones, α-blockers or calcium-channel inhibitors are usually recommended, such as tamsulosin, silodosin and nifedipine (Hollingsworth et al., 2006; Turk et al., 2016). Yet, it seems that this class of medicine can only increase passage in larger stones (>5 mm) rather than smaller stones (Hollingsworth et al., 2016). Furthermore, these drugs increase the risk of orthostatic hypotension, which can be particularly problematic in older subjects (Gottlieb et al., 2018). Presently, numerous drugs have been studied for the management of urolithiasis, allopathic medicine are still limitedly available (Ahmad et al., 2021).

The total flavonoids of *Desmodium styracifolium* (TFDS) are the flavonoid-enriched natural extracts purified from Desmodii Styracifolii Herba, which is a traditional herbal medicine for treating urolithiasis in China (Hou et al., 2018). It was reported that TFDS can significantly reduce crystalluria and improve the impaired renal function in the urolithiasis rat models (Zhou et al., 2018). The results of a randomized, double-blind, multi-center clinical trial performed in China (CTR20171265) showed that TFDS had a significant effect on the efficiency of stone removal and improvement of TCM Symptoms. The Capsule of TFDS was approved by National Medical Products Administration (NMPA) in China for the treatment of urinary calculi (https://www.nmpa.gov.cn/yaowen/ypjgyw/20220915152305161.html) in 2022 (Z20220003).

The main active constituents of TFDS are the flavone glycosides, including schaftoside, vicenin-1, vicenin-2, vicenin-3, et al. (Li et al., 2022). Schaftoside is the marker component of *Desmodium styracifolium* in Chinese Pharmacopeia (2020 edition). An *in vivo* study demonstrated that the plasma concentration of schaftoside was the highest among these components after oral intake of TFDS (Li et al., 2022). Previous studies indicated that schaftoside could protect against cholesterol gallstone and calcium oxalate kidney stone formation (Liu et al., 2017; Liu et al., 2020). To some extent, schaftoside is the representative compound of TFDS. Although the pharmacokinetic (PK) characteristics of schaftoside following administration of TFDS have been studied in animals, the PK profiles of it in humans remain unknown (Li et al., 2022).

The crystalline deposits in kidney tubular cells lead to acute or chronic tubular injury and interstitial fibrosis, which contributes to a progressive chronic kidney disease (CKD) (Khan et al., 2016). However, PK studies in the CKD patients are difficult to conduct due to ethical and logistic challenges. Physiologically based pharmacokinetic (PBPK) modeling is a mathematical modeling technique to describe and predict drug disposition in various populations (Jones and Rowland-Yeo, 2013). By integrating population-specific physiologic parameters with drug-specific physicochemical and pharmacokinetics information, PBPK models are increasingly used for the prediction of drug distribution, drug-drug interactions (DDI), transporter evaluation, and extrapolation of drug exposure in age-specific or special subgroups of patients (Jones et al., 2009; Maharaj and Edginton, 2014; Reig-Lopez et al., 2021). However, the application of PBPK modeling for the active ingredients of herbal medicine was limitedly reported. Thus, the aim of this study was to establish a PBPK model of schaftoside after oral administration of TFDS in rats and humans, and to extrapolate the adult PBPK model to several subpopulations including elderly and renally impaired population for the prediction of plasma concentration-time profiles of schaftoside.

## 2 Materials and methods

### 2.1 Chemicals and reagents

TFDS Capsules (0.2 g/capsule), containing 133 mg of TFDS extract with 8.5 mg schaftoside per capsule, and the TFDS APIs were supplied by Humanwell Healthcare (Group) Co., Ltd. The reference standards of schaftoside and sulfamethoxazole were provided by Chinese National Institutes for Food and Drug Control (Beijing, China). Midazolam was supplied by Toronto Research Chemicals INC (Toronto, Canada). D-glucose-6-phosphate disodium salt hydrate, glucose-6-phosphate dehydrogenase, β-nicotinamide adenine dinucleotide phosphate sodium salt hydrate, and midazolam were the products of Sigma-Aldrich Co. (St. Louis, United States). The liver microsomes of rat and human were purchased from Corning Gentest (Woburn, MA, United States). High-performance liquid chromatography (HPLC) grade acetonitrile, methanol and formic acid were supplied by Merck KGaA (Darmstadt, Germany). Ultrapure water was prepared by a Milli-Q water system (Millipore, United States) in our own lab. All the other reagents and solvents were commercially available.

### 2.2 Software

Pharmacokinetic parameters of schaftoside were calculated by non-compartmental model analysis using Phoenix WinNonlin software (version 8.3, Certara Corporation, United States). The establishment of PBPK models was used PK-Sim v9.0 which is part of the Open Systems Pharmacology suite (www.open-systems-pharmacology.org). The optimization and sensitivity analysis of model input parameters were carried out by PK-Sim. *Python* Software (Version 3.7, https://www.python.org/) was used for graph plotting.

### 2.3 Metabolic stability in liver microsomes

The *in vitro* metabolic stability of schaftoside was investigated in rat and human liver microsomes. All incubations were conducted in triplicate at 37°C with a final volume of 400 μL. The incubation mixture contained schaftoside (1 μM, 10 μM), liver microsomes (0.5 mg protein/mL), and NADPH-generating system (3.3 mM glucose 6-phosphate, 1.3 mM NADP^+^, 4 mM MgCl_2_, and 0.4 U/mL glucose 6-phosphate dehydrogenase) in Tris-HCl buffer (50 mM, pH 7.4). The mixture was incubated for 5 min at 37°C before the addition of NADPH to initiate the reaction. The reaction was terminated by the addition of a 2-fold volume of ice-chilled acetonitrile at specific time points (0, 0.5, 1.0 and 1.5 h). Then the mixture was vortexed for 1 min and centrifuged at 12,000 rpm for 10 min at 4°C. The supernatant was stored at −20°C until HPLC-MS/MS analysis. Midazolam (5 uM) was used as a positive control and the total organic solvent concentration did not exceed 1% (v/v) in the experiment.

### 2.4 Pharmacokinetic and excretion studies in rats

Male Sprague-Dawley rats (6 weeks, 200–220 g) were purchased from Vital River Experimental Animal Co., Ltd (Beijing, China). The rats were housed under standard laboratory conditions at room temperature (20°C–25°C) with relative humidity (50–60%) and 12-h day/night rhythm cycle. Rats were fasted overnight with free access to water before the experiment. The experimental protocols were approved by the Animal Care and Welfare Committee of Shanghai University of Traditional Chinese Medicine.

For determination of pharmacokinetic characteristics of schaftoside in TFDS, rats were either administered a single intravenous dose of 1.2 mg/kg of schaftoside solution (5% ethanol +5% PEG400 in saline) or an oral dose of 50, 100, or 200 mg/kg TFDS suspension (0.5% CMC-Na, equivalent to 3.03, 6.06, 12.12 mg/kg schaftoside). The oral dose of TFDS was chosen based on the results of our previous pharmacological experiments and relevant literature (Zhou et al., 2018), which showed that the effective dose range of TFDS was between 50 and 400 mg/kg. Due to the low oral bioavailability of schaftoside (<5%), ten percent of the amount of schaftoside in the high-dose group of TFDS (12.12 mg/kg) was selected as the intravenous dose.

Six rats were used per experimental group in the pharmacokinetic studies. Blood samples (approximately 0.15 ml) were collected in 1.5 ml heparinized tubes pre-dose (0 h), and 0.033, 0.083, 0.167, 0.333, 0.50, 0.75, 1, 2, 3, 4, 6, 8, and 12 h post-dose for intravenous dosing, and pre-dose (0 h), and 0.083, 0.25, 0.50, 0.75, 1, 1.5, 2, 3, 4, 6, 8, 12, 24 h post-dose for oral administration. Plasma was prepared by centrifugation at 4,000 rpm for 10 min at 4°C, and samples were stored at −80°C until analysis.

For the excretion studies of schaftoside, six rats received 1.2 mg/kg of schaftoside solution through tail vein injection. Three rats were placed in metabolic cages individually and urine was collected during the following intervals: 0–4, 4–16, 16–24, 24–36, 36–48 h post dosing. Another three rats were anesthetized and cannulated with PE-10 polyethylene tubing for the collection of bile. Bile samples were collected during the following intervals: 0–2, 2–4, 4–16, 16–24, 24–36, 36–48 h post dosing. In addition, urine was collected following the same protocols after six rats were orally administered 100 mg/kg TFDS suspension. The volumes of urine and bile samples were measured and recorded. Renal clearance (CLr) was calculated through the determination of the total amount of drug excreted in urine (Ae) and drug exposure in plasma (AUC_0-t_) after a single administration, which was described as CLr = Ae/AUC_0-t_.

### 2.5 Clinical pharmacokinetic studies in healthy volunteers

This clinical study was an open-label, single-center, single- and multiple-dose study, which was conducted at Longhua Hospital, Shanghai University of Traditional Chinese Medicine. The study protocol was approved by the Institutional Review Board of Longhua Hospital (Approved Number 2019LCSY069) and registered at Chinese Clinical Trials Platform (CTR20192424). All subjects provided written informed consent before this study. The trial strictly complied with the Declaration of Helsinki and the International Conference on Harmonization (ICH) Good Clinical Practice Guidelines.

Four healthy volunteers (two men and two women) aged 18–45 years with body mass index (BMI) of 19–24 kg/m^2^ and body weight ≥50 kg were enrolled into this study. Participants were excluded for any abnormal screening laboratory results, HIV/HBV/HCV infection, and clinically significant abnormality on physical examination or electrocardiogram. Additional exclusion criteria included a history of hypersensitivity to study drugs or excipients; drug abuse within the past 12 months or alcohol abuse within the past 6 months; pregnancy; vaccination within 4 weeks; intake of prescription or non-prescription medications within 2  weeks before hospital admission.

The subjects were asked to fast for at least 10 h before the first oral administration. The subjects were orally administered of TFDS Capsules once a day for day 1 and day 6 (0.6 g/day), and three times a day from day 2 to day 5 (0.6 g q 8 h). TFDS Capsules were administered at 08: 00, 16: 00, 00: 00 from day 2 to day 5, and at 08:00 on day 1 and day 6. Venous blood samples (∼4 ml) were collected into K_2_EDTA vacuum tubes on day 1 and day 6 at pre-dose (0 h) and 0.25, 0.5, 1, 1.5, 2, 2.5, 3, 4, 5, 6, 7, 8, 12, 24 h post-dose. Blood trough samples were collected before the first dose (08: 00) on day 2 to day 5. Plasma was separated from the blood samples by centrifugation at 3,500 rpm for 10 min at 4°C, and stored at −80°C until analysis.

### 2.6 HPLC-MS/MS analysis

#### 2.6.1 Sample pretreatment

For the rat sample pretreatment, an aliquot of 50 μl plasma/urine/bile sample was spiked with 10 μL of sulfamethoxazole solution (500.0 ng/ml, internal standard) and 240 μl methanol. Then the mixture was vortexed for 1 min and centrifuged at 12,000 rpm for 10 min at 4°C. The supernatant was collected and analyzed by HPLC-MS/MS.

For the human sample pretreatment, an aliquot of 100 μl plasma sample was spiked with 10 μl of sulfamethoxazole solution (100.0 ng/ml) and 500 μl mixed solvent (acetonitrile: methanol = 3:2, v/v). Then the mixture was vortexed for 1 min and centrifuged at 12,000 rpm for 10 min at 4°C. The supernatant was dried for 3 h at 30°C using a vacuum centrifugal concentrator (Concentrator plus, Eppendorf AG, Hamburg, Germany). The residues were reconstituted with 100 μl of 50% methanol and centrifuged again. The supernatant was analyzed by HPLC-MS/MS.

#### 2.6.2 Instrumentation and chromatographic conditions

The plasma concentration of schaftoside was determined using a liquid chromatography tandem mass spectrometry method (HPLC-MS/MS) as previously reported (Li et al., 2022). Briefly, the HPLC-MS/MS system was comprised of an Agilent 1260 HPLC system and a tandem mass spectrometer (AB SCIEX QTRAP^®^5500, Canada). The chromatographic separation was performed on an ACQUITY UPLC HSS T3 column (1.8 μm, 2.1 mm i. d. × 50 mm; Waters, United States), and a gradient elution was adopted using water containing 0.1% (v/v) formic acid (A) and methanol (B) as the mobile phase. The following gradient program was used: 0–1.0 min, 10% B; 1.0–4.0 min, 10–30% B; 4.0–6.0 min, 30–50% B; 6.0–10.0 min, 50% B; 10.0–11.0 min, 50–10% B; 11.0–15.0 min, 10% B. Mass spectrometry was performed under negative electrospray ionization condition. The selected mass transitions for schaftoside and its internal standard sulfamethoxazole were m/z 563.2→353.2 and m/z 252.0→156.0, respectively.

The calibration curves for schaftoside in rat and human plasma samples were linear over ranges from 0.5 to 200 ng/ml and 0.05–10 ng/ml, respectively. The analytical methods were well validated in this study. The intra-day and inter-day precision and accuracy values met the acceptance criteria ( ± 15%) according to the FDA and NMPA guidelines.

### 2.7 Establishment of PBPK models

**FIGURE 1:**
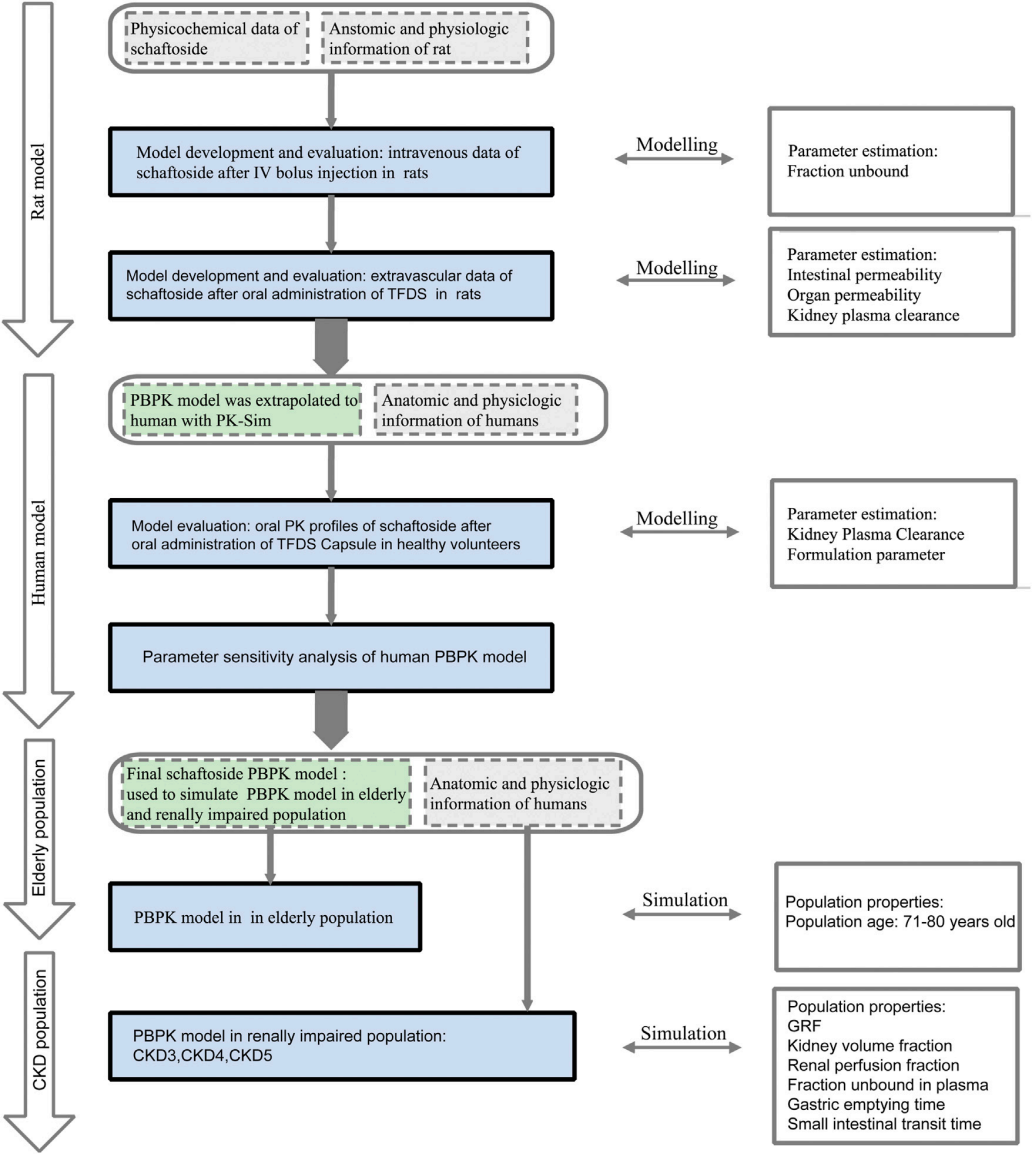
Schaftoside PBPK model-building workflow in rats and humans after oral administration of TFDS. The overall scheme included development, verification, and application.

The overall scheme of the PBPK model-building workflow including development, verification, and application is listed in Figure 1. Briefly, the PBPK model of schaftoside was firstly built for rats, which was subsequently evaluated with experimental data to promote confidence in the parametrization of the model, and then the model was scaled to humans. The basic physicochemical and biopharmaceutical parameters of schaftoside were mainly gained from the literature, online databases and PK-Sim^®^ software prediction and optimization (Table 1) (Mendez et al., 2019; Shen et al., 2021). The default physiological parameters of animals and humans in software were modified to represent our experiments such as weight, age and gender. All relevant anatomical and physiological parameters for rat and human including tissue volume, blood flow and vascular permeability were contained in the integrated database within the software.

**TABLE 1:** Parameters used to establish the PBPK models of schaftoside in rat and human.

Parameter	Value	Unit	Source	Description
Mwt	596.494	g/mol	ZINC000084817379^a^	Molecular weight
LogP	−2.427	—	ZINC000084817379^a^	Lipophilicity
Acidic_pKa	6.35	—	CHEMBL532274^b^	First acid dissociation constant
Solubility (PH-7)	0.02	g/L	Calculated by XLOGS^c^	Solubility
f_u_	26.65%	%	Optimized	Fraction unbound
Intestinal permeability	1.08*1E-4	dm/min	Optimized	Transcellular intestinal permeability
Organ permeability	2.98*1E-6	cm/s	Optimized	Organ permeability
Formulation (rat)	Suspension	—	—	—
Kidney Plasma Clearance (rat)	2.57*1E-3	L/min/kg	Optimized	Kidney Plasma Clearance
Dis time (rat)	0.92	Min	Optimized	Dissolution time (50% dissolved)
Dis shape (rat)	1.08*1E-3	—	Optimized	Shape parameter of Welbull function
Formulation (Human)	Capsule	—	—	—
Kidney Plasma Clearance (human)	1.24*1E-3	L/min/kg	Optimized	Kidney plasma clearance
Dis time (human)	8.16	Min	Optimized	Dissolution time (50% dissolved)
Dis shape (human)	3.74*1E-4	—	Optimized	Shape parameter of Welbull function

^a^ Available at https://zinc.docking.org/substances/ZINC000084817379/

^b^ Literature report (Mendez et al., 2019).

^c^ Available at http://www.sioc-ccbg.ac.cn/?p=42&software=xlogs

By utilizing the plasma concentration data of schaftoside after a bolus intravenous injection of this chemical, the elimination PK model of schaftoside in rats was firstly established. After getting accurate distribution and elimination parameters, a PBPK model for extravascular administration was further developed. The parameters related with absorption, distribution and metabolism/excretion were fixed and modified (Kuepfer et al., 2016). In this study, the plasma concentration data of schaftoside after an oral dose of 100 mg kg^−1^ TFDS suspension were used to perform and optimize the extravascular PBPK model. The plasma concentration profile of schaftoside obtained from the dose groups of 50 and 200 mg/kg were used for the validation of PBPK model.

For the development of human PBPK model for schaftoside after oral administration of TFDS Capsules, the formulation of drug was changed from suspension to capsule, so we reset the parameters of dissolution time and shape. We selected the pre-existing individual option in PK-Sim^®^ software which was a 28-year-old Japanese male with 56.35 kg of body weight and 162.1 cm of height. In order to assess the accuracy of the extrapolation, the predicted and observed data obtained from a clinical PK study performed in healthy volunteers were compared.

### 2.8 PBPK model validation

The predictive performance of PBPK model was mainly evaluated by visual predictive checks, fold error of each concentration-time points and main PK parameters such as C_max_ and AUC_0-t_. Agreement between the predicted and observed profiles was evaluated by comparing the predicted mean concentration-time profiles with mean observed data in goodness-of-fit plots. The observed data were obtained from our experimental data and previous literature reports (Li et al., 2022).

The fold error of each point (FE_i_), average fold error (AFE) and absolute average fold error (AAFE) were three commonly used test criteria to evaluate the accuracy of models (Tan et al., 2021). FE_i_ showed the predictive accuracy of data at time point *i*, as calculated in Eq. 1. AFE showed whether the predicted profile overestimated or underestimated the observed values, as calculated in Eq. 2 (Huang and Isoherranen, 2020). AAFE indicated the absolute error from the observed values, as calculated in Eq. 3 (Rasool et al., 2021).

$$FE_{i}=\frac{Predicted_{i}}{Observed_{i}}$$ (1)

$$AFE=10^{\frac{1}{n}*\sum \log\left(\left|\frac{Predicted_{i}}{Observed_{i}}\right|\right)}$$ (2)

$$AAFE=10^{\frac{1}{n}*\sum \left|\log\left(\frac{Predicted_{i}}{Observed_{i}}\right)\right|}$$ (3)

In which *predicted*_*i*_ is the predicted concentration at time point *i*, *observed*_*i*_ is the observed concentration at time point *i*, *n* is the number of time points. For each point, it can be considered as a successful simulation if the *FE*_*i*_ ranges from 0.3 to 3 and the *AFE* and *AAFE* both range from 0.5 to 2.

The fold error (FE) of PK parameters (C_max_ and AUC_0-t_) was also commonly used to evaluate the accuracy of prediction (Eq. 4). A good prediction model is achieved when FE is between 0.5 and 2 (Ke et al., 2022).

$$FE=\frac{Predicted\;values\;of\;parameter}{Observed\;values\;of\;parameter}$$ (4)

### 2.9 Parameter sensitivity analysis

Parameter sensitivity analysis was performed using PBPK model established in human with the sensitivity analysis tool provided with the PK-Sim^®^ software (Yellepeddi and Baker, 2020). C_max_ and AUC_0-t_ of schaftoside were evaluated to find out the key factors that influenced the simulated schaftoside plasma concentration-time profiles.

Sensitivities of the PBPK models were calculated as the relative changes of the predicted PK parameter of schaftoside to the relative variation of model input parameters. The analyses were investigated with a relative perturbation of initial input values of 10%. Sensitivity analysis to a model parameter was calculated as follows:

$$S=\frac{\Delta PK}{\Delta p}\cdot \frac{p}{PK}$$ (5)

where *S* is the sensitivity of the PK parameter to the examined model parameter, *∆PK* is the difference between the values of PK values in the new simulation and the original simulation. *PK* is the simulated value with the original parameter value, *p* is the original input parameter and *∆p* is the change of the *p*.

The sensitivity value of −1.0 shows that a 10% increase of the parameters causes a 10% decrease of the PK parameter values, and a sensitivity of +0.5 implies that a 10% increase of the parameters causes a 5% increase of the PK parameter values (Kovar et al., 2020).

### 2.10 PBPK model prediction in elderly and renally impaired population

The PBPK model in adults was scaled to elderly individuals by adjusting age to 70 years old. The changes of whole-body anatomical and physiological parameters related based on age were induced using the in-built calculation methods and integrated database in PK-Sim^®^ software. Population simulations were used to quantify the relationship between PK parameter and inter-individual variability. It was designed in PK-Sim to create a virtual population using manual settings for the number of simulated subjects, sex ratio, age range, *etc.* Simulations of elderly population were conducted by setting with 100 subjects with 50% male, age ranging from 71 to 80 years based on elderly individual parameters.

For the renal impairment simulations, 100 subjects with 50% male, age ranging from 18 to 40 years with demographic properties according to the clinical study data were created. CKD is the most prevalent form of renal impairment. In this study, we used three levels of CKD, classified based on the GFR ranges 30–60, 15–30, and <15 ml/min/1.73 m^2^ (Yoon et al., 2019). PBPK models for populations with “healthy” “CKD *stage3*” “CKD *stage4*” “CKD *stage5*” were established, respectively. The final virtual healthy individual models were replaced with individuals whose parameter sets were adapted for renal impairment meanwhile the parameters of drug were left unchanged. The whole-body anatomy and physiology in patients throughout the progressive stages of CKD such as kidney volume fraction, renal perfusion fraction, fraction unbound in plasma, gastric emptying time and small intestinal transit time were changed according to literatures (Malik et al., 2020). Changes in the physiological parameters, resulting from decreased renal function, were listed in Table 2. Single-dose (0.6 g or 1 g) and multi-dose simulations (0.6 g q 8 h or 1 g q 8 h) of TFDS Capsules were performed in patients with different stages of renal impairment. The influence of renal impairment on plasma exposure of schaftoside was conducted *via* visual inspection of the plasma concentration time profile and by comparison of PK parameters (Cui et al., 2021).

**TABLE 2:** Categorical parameters in healthy adults and renally impaired patients with different CKD stages (age range: 18–40, 50% female).

	Fraction of Healthy Values (normal Coefficient of Variation %)			
Parameter	Healthy	Stage 3	Stage 4	Stage 5
	110.57 ml/min/1.73 m^2^	30–60 ml/min/1.73 m^2^	15–30 ml/min/1.73 m^2^	<15 ml/min/1.73 m^2^
GRF	110.57	60.00	30.00	15.00
Kidney volume fraction	1.00	0.81	0.61	0.51
renal perfusion fraction	1.00	0.55	0.36	0.29
Fraction unboun in plasma	1.00	1.07	1.16	1.55
Gastric emptying time	1.00	1.00	1.60	1.60
Small intestinal transit time	1.00	1.00	1.40	1.40
Large intestinal transit time	1	1	1	1

## 3 Results

### 3.1 *In vitro* metabolism by liver microsomes

**FIGURE 2:**
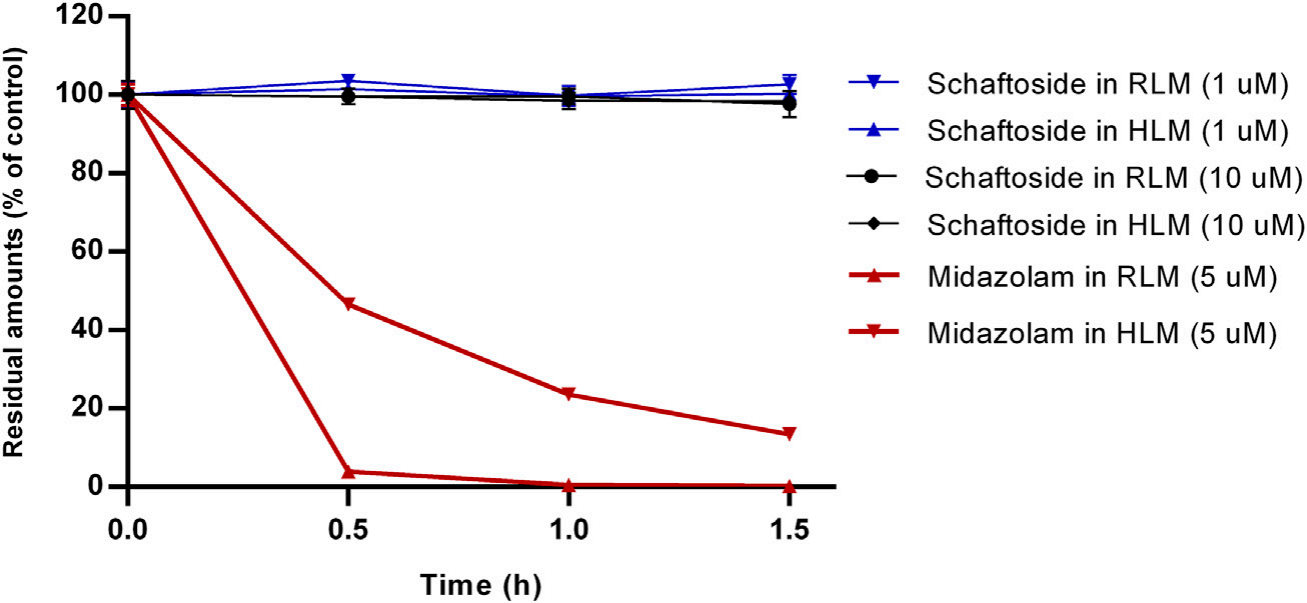
*In vitro* metabolism of schaftoside in rat liver microsomes (RLM) and human liver microsomes (HLM). Midazolam, a representative substrate of CYP3A4/5 enzyme in liver microsomes, was used a positive control of this study.

The results of microsomal metabolic stability of schaftoside are shown in Figure 2. After 1.5 h incubation with rat liver microsomes, the remaining amount of schaftoside accounted for 102.66 ± 2.41% (C_0_ = 1 μM) and 97.66 ± 3.30% (C_0_ = 10 μM) of the initial amounts. Similar to the results above, the residual schaftoside amounts in human liver microsomes were 100.25 ± 1.04% (C_0_ = 1 μM) and 98.36 ± 1.34% (C_0_ = 10 μM) after 1.5 h. In contrast, midazolam (5 μM), a representative substrate of CYP3A4/5 enzyme, was metabolized quickly in rat and human liver microsomes, in which the residual amounts of midazolam were 0.24 ± 0.05% and 13.47 ± 0.75%, respectively. These results indicated schaftoside was poorly metabolized by the liver microsomes.

### 3.2 Pharmacokinetic and excretion studies in rat

**FIGURE 3:**
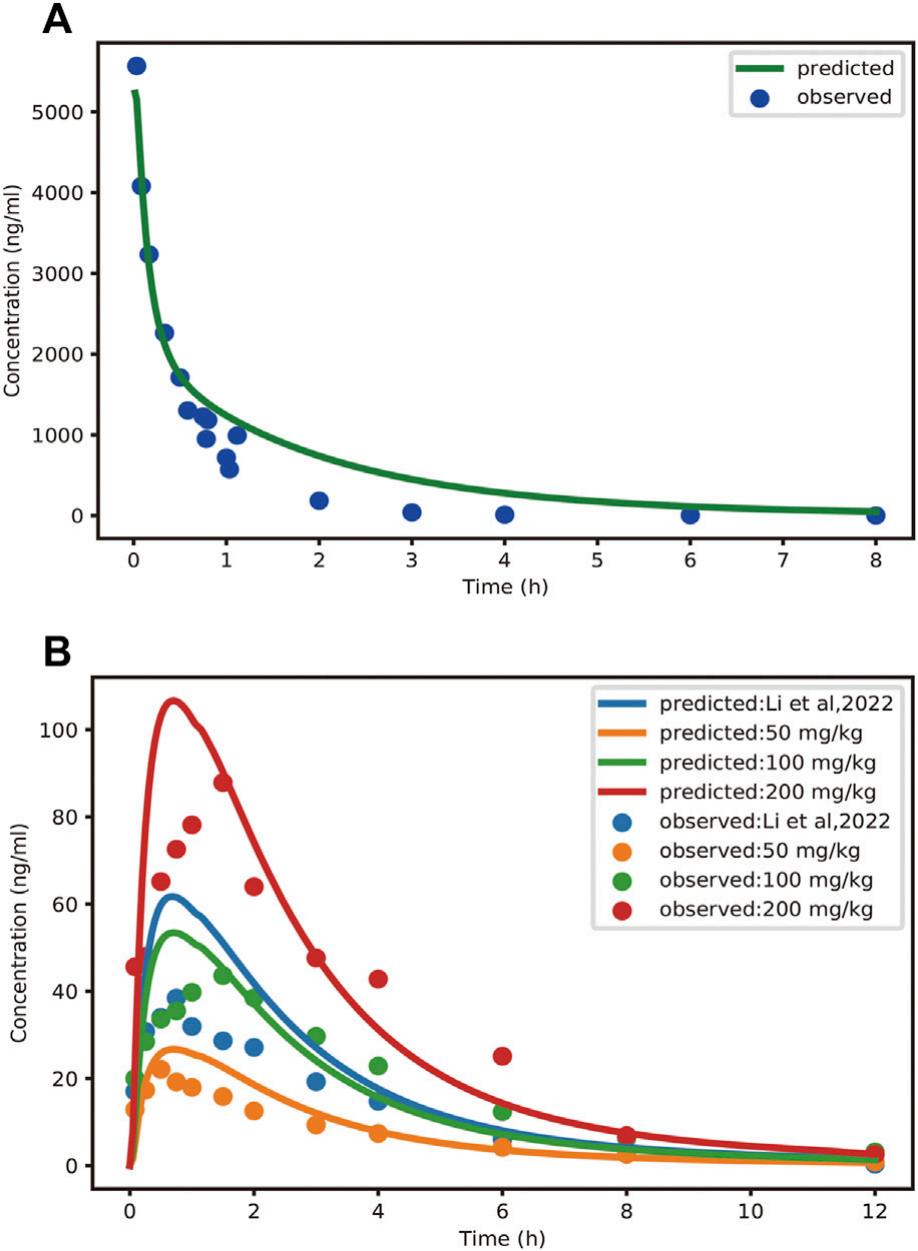
Observed and PBPK-model-predicted plasma concentration-time profiles of schaftoside after intravenous injection of schaftoside solution **(A)** and oral administration of TFDS suspension **(B)**. Solid circles represent observed mean data, and solid lines represent the profiles predicted from the PBPK model in PK-Sim. Doses were 1.2 mg/kg (iv) and 50, 100, 200 mg/kg (po), respectively. Additionally, the dose of TFDS in the previous report (Li et al., 2022) is 100 mg/kg.

The mean concentration-time profiles and pharmacokinetic parameters of schaftoside in rats are presented in Figure 3 and Table 3, respectively. After a single intravenous injection at 1.2 mg/kg dose, the mean maximum plasma concentration (C_max_) of schaftoside in rats reached 5567.22 ng/ml. Schaftoside was quickly eliminated from the blood circulation with a terminal half-life (t_1/2_) of 0.64 h. Meanwhile, schaftoside exhibited a low volume of distribution in rats following the intravenous administration. After oral administration of TFDS suspension at 50, 100 and 200 mg/kg doses, the plasma concentration of schaftoside reached its peak at 0.25–1.50 h. Schaftoside plasma exposures were generally proportional to the administered doses. The C_max_ and AUC_0-t_ values of schaftoside in low, medium, high groups were 23.17, 46.25, 91.75 ng/ml and 76.39, 204.75, 357.09 hng/mL, respectively. In addition, no significant between-group differences were observed in t_1/2_, Vz_F and CL_F variables.

**TABLE 3:** Pharmacokinetic parameters of schaftoside in rats after intravenous bolus of schaftoside solution or oral administration of TFDS suspension (n = 6). Data are presented as the mean ± standard deviation except for T_max_ as median (range).

Drug	Dose (mg/kg)	C_max_ (ng/ml)	t_max_ (h)	AUC_0-t_ (h·ng/mL)	t_1/2_ (h)	Vz_F (L/kg)	CL_F (L/h^/^kg)
Schaftoside (iv)	1.2	5567.22 ± 1007.46	—	2776.80 ± 449.16	0.64 ± 0.10	0.41 ± 0.10	0.44 ± 0.07
TFDS (po)	50^a^	23.17 ± 5.07	0.50 (0.25–1.5)	76.39 ± 20.26	2.22 ± 0.47	132.72 ± 26.39	39.00 ± 9.76
TFDS (po)	100^b^	46.25 ± 15.94	1.00 (0.50–1.50)	204.75 ± 59.50	2.18 ± 0.53	114.70 ± 44.07	30.22 ± 9.87
TFDS (po)	200^c^	91.75 ± 18.59	1.25 (0.50–1.50)	357.09 ± 123.71	2.03 ± 0.09	105.42 ± 29.09	35.86 ± 9.31

^a,b,c^Equivalent to 3.03, 6.06, 12.12 mg/kg schaftoside, respectively.

**FIGURE 4:**
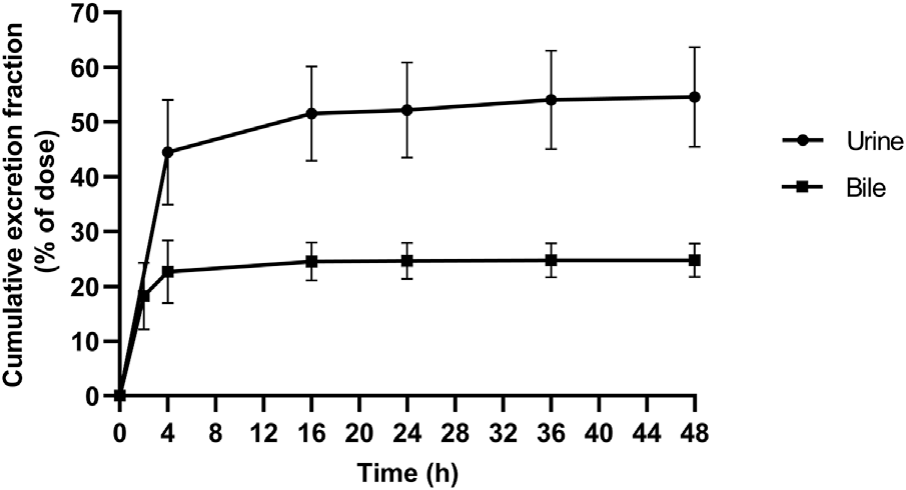
Cumulative excretion of schaftoside into urine and bile after an intravenous administration in rats.

In the current study, the excretion pathways of schaftoside in rats were firstly investigated after an intravenous injection of schaftoside solution. After drug administration, the total cumulative urinary and biliary excretion of schaftoside accounted for 54.59 ± 9.11% and 24.78 ± 3.07% of the dose, respectively (Figure 4). The calculated renal clearance was 50.67 ± 11.67 ml/h (Table 3; Figure 3, Figure 4). Moreover, the cumulative urinary amounts of schaftoside were measured in rats after oral administration of TFDS, and the results showed 57.56 ± 5.88% of the absorbed schaftoside were was excreted unaltered through kidney. The results indicated that the urinary and biliary excretions of the parent form are the major elimination routes of schaftoside.

### 3.3 Clinical pharmacokinetic studies in healthy volunteers

In the current study, 29 subjects signed informed consent, and 4 subjects met all the enrollment criteria and entered the clinical trial. The primary reasons for exclusion included withdrawal of consents, electrocardiographic abnormality, and liver or kidney function alterations. All participants completed this study. Demographic data on the participants (4 subjects) showed the mean age was 28 years old (range: 23–32 years old) and 50% were female. The body mass index ranged from 20.8 to 21.9 kg/m^2^ and the median index was 21.5 kg/m^2^. No serious adverse events related to the drug were observed during the clinical trial.

**FIGURE 5:**
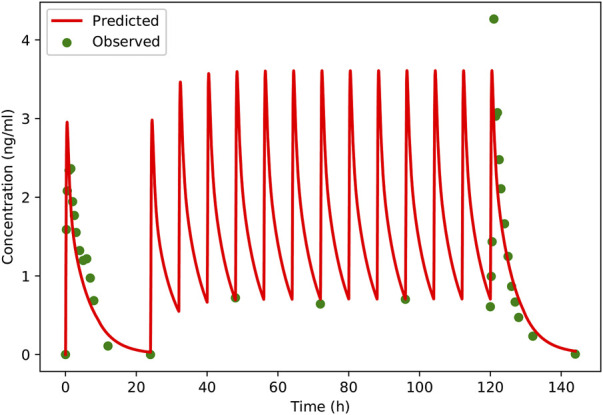
Predicted and observed plasma concentration-versus-time profiles of schaftoside after single- and multiple-dose of TFDS Capsules to healthy volunteers (two male and two female). Clinically observed data are shown as dots; solid lines indicate the model prediction.

The pharmacokinetic parameters of schaftoside in human subjects following single- and multiple-dose administration of TFDS Capsules are shown in Table 4, and the concentration-time profiles are presented in Figure 5. After the oral dose of TFDS Capsules on day 1 (0.6 g), the maximum concentration of schaftoside was reached within 1.0–1.5 h. The mean C_max_ and AUC_0-t_ values were 2.43 ng/ml and 13.19 hng/mL, respectively. The plasma concentrations were close to the detection limit (0.05 ng/ml) at 12 h after dosing. In the multiple-dose part, the trough concentration of schaftoside was determined for 3 consecutive days. The mean trough concentrations on day 3, 4 and 5 were 0.72, 0.64, 0.70 ng/ml, respectively. The values were analyzed by one-way ANOVA, and no significant differences were observed (*p* > 0.05). Therefore, steady-state of schaftoside concentrations appeared to be achieved after three doses of TFDS Capsules. Compared with the first-dose on day 1, the C_max_ and AUC_0-t_ values of schaftoside on day 6 were slightly elevated. The R_Cmax_ (ratio of C_max6_ *versus* C_max1_) and R_AUC_ (ratio of AUC_last6_ *versus* AUC_last1_) were 1.64 and 1.23, respectively. The accumulation index of schaftoside was 1.12 in this study.

**TABLE 4:** Pharmacokinetic parameters of schaftoside in human subjects (n = 4) after single- and multiple-dose of TFDS Capsules. Data are presented as the mean ± standard deviation except for T_max_ as median (range).

Variable	Units	Day 1	Day 6
C_max_	ng/mL	2.43 ± 1.00	4.32 ± 3.54
T_max_	h	1.25 (1.00–1.50)	1.50 (1.00–2.00)
AUC_0-t_	h·ng/mL	13.19 ± 4.16	15.62 ± 5.77
t_1/2_	h	1.62 ± 0.21	2.41 ± 0.86
Cl_F	L/h	2048 ± 655	—
Vz_F	L	4740 ± 1530	—
Vss	L	—	6845 ± 2668
C_avg_	ng/mL	—	1.69 ± 0.65
AUC__TAU_	h·ng/mL	—	13.55 ± 5.19

### 3.4 PBPK modelling and evaluation in rat and human

In this study, the PBPK model was initially optimized and validated in rats by using the obtained preclinical data. The observed and predicted plasma concentration-time profiles of schaftoside in rat are presented in Figure 3, and the predicted and observed values of PK parameters such as C_max_ and AUC_0-t_ are shown in Table 5. As shown in Figure 3, the predicted plasma concentration profiles of schaftoside were in close concordance with observed data except that the predicted maximum plasma concentration was generally higher than the observed values in the oral intake groups. The fold error (FE) of C_max_ ranged from 0.94 to 1.41, and FE of AUC_0-t_ was between 0.87 and 1.65, which all fell within the 2-fold acceptance criterion (Table 5). These results indicated that the PBPK model successfully predicted schaftoside pharmacokinetics in rats.

**TABLE 5:** Predicted and observed pharmacokinetic parameters of schaftoside in rats. The accuracy of the prediction is expressed as fold error (the ratio of predicted to observed).

	1.2 mg/kg (schaftoside, iv)		50 mg/kg (TFDS, po)^a^		100 mg/kg (TFDS, po)^b^		200 mg/kg (TFDS, po)^c^		100 mg/kg (TFDS, po)^d^	
	C_max_ (ng/mL)	AUC_0-t_ (h·ng/mL)	C_max_ (ng/mL)	AUC_0-t_ (h·ng/mL)	C_max_ (ng/mL)	AUC_0-t_ (h·ng/mL)	C_max_ (ng/mL)	AUC_0-t_ (h·ng/mL)	C_max_ (ng/mL)	AUC_0-t_ (h·ng/mL)
Observed	5567.22	2776.8	23.17	76.39	46.25	204.75	91.75	357.09	43.86	127.56
Predicted	5230.70	4587.42	26.69	89.09	53.41	178.26	106.64	356.62	61.72	201.76
FE	0.94	1.65	1.15	1.17	1.15	0.87	1.16	1.00	1.41	1.58
AFE	—		1.03		0.86		1.02		1.51	
AAFE	—		1.35		1.47		1.33		1.64	

^abc^ Equivalent to 3.03, 6.06, 12.12 mg/kg schaftoside, respectively.

^d^ Data from literature report (Li et al., 2022).

A PBPK model was constructed for the healthy adults based on the rat model, and then the model was verified using clinical PK data. The predicted and observed plasma concentration-time profiles of schaftoside in human are presented in Figure 5. As shown in the figure, the predicted plasma profiles are in close agreement with the observed data. The pharmacokinetic parameters of schaftoside were calculated for day 1 and day 6. The predicted C_max_ and AUC_0-t_ values were 1.38- and 1.05-fold of the observed data in the single-dose section (day 1), while the predicted values for C_max_ and AUC_0-t_ of in the multi-dose regimens were 0.95 and 1.18 (day 6). The fold error of PK parameters met the acceptance criteria of 2-fold.

**FIGURE 6:**
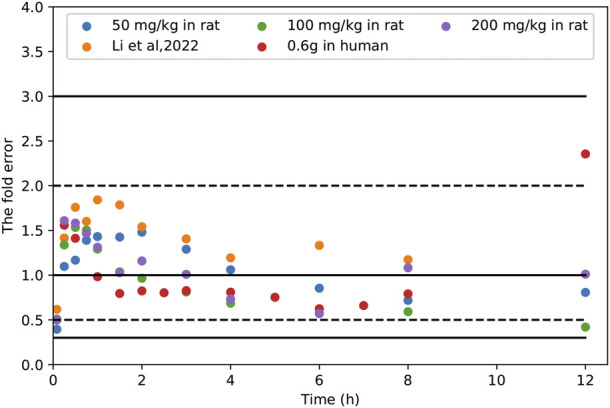
The fold errors of all the predicted and observed concentration points (FE_i_) in Figure 3 and Figure 5. The data was calculated by Predicted_*i*_/Observed_*i*_. The 2-fold and 3-fold errors are indicated by dashed and solid black lines, respectively.

In order to further evaluate the accuracy of the PBPK models in rat and human, each point of the models was validated by FEi. As shown in Figure 6, most of the FEi values were close to 2, but there were a few outliers. The FE_i_ value of time at 12 h of model in rat simulating 100 mg/kg TFDS (literature report) was without 3-fold error (FE_i_ = 4.47. These may be caused by experiment errors in the blood sampling or detection for the last time point (Tan et al., 2021). In addition, the AFE and AAFE values for models in rat and human were all within 2-fold error (Table 5). These results indicated the PBPK models in rat and human are accurate and reliable.

### 3.5 Parameter sensitivity analysis

**FIGURE 7:**
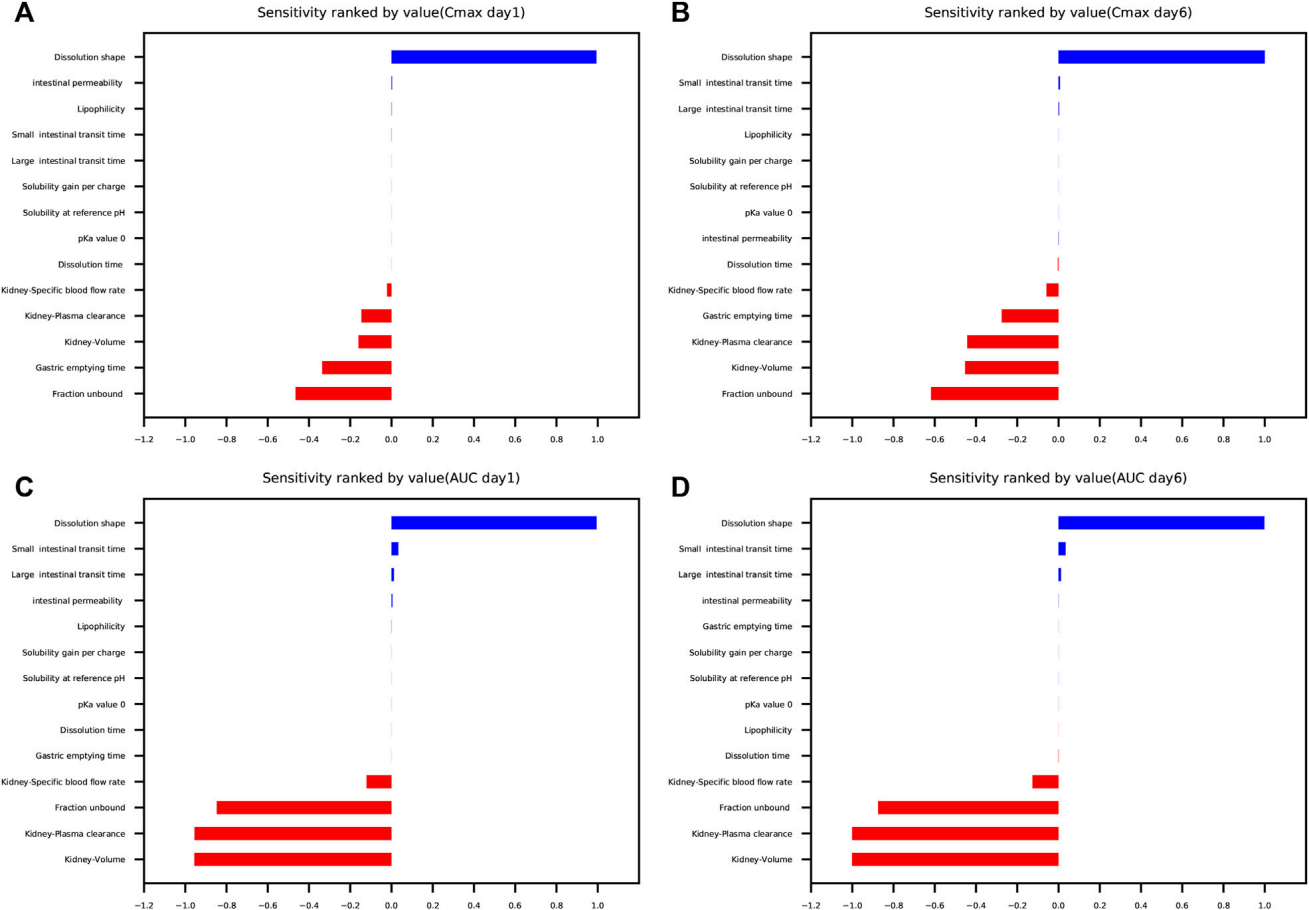
Parameter sensitivity analysis performed on the human PBPK model with PK-Sim^®^. Sensitivity was measured as the relative changes of C_max_ on day 1 **(A)**, C_max_ on day 6 **(B)**, AUC_0-t_ on day 1 **(C)**, AUC_0-t_ on day 6 **(D)** of schaftoside in the clinical study. Variation range was 0.1 with maximum number of steps = 2.

The results of parameter sensitivity analysis performed on the human PBPK model are demonstrated in Figure 7. Figures 7A,B suggested that the predicted C_max_ values of schaftoside both on day 1 and day 6 were most sensitive to changes in the dissolution shape of drugs. The parameter was also sensitive to changes in fraction unbound, gastric emptying time, kidney-volume, kidney-plasma clearance, and kidney-specific blood flow rate. Whereas, C_max_ was insensitive to the following factors: pKa value 0, solubility at reference pH, solubility gain per charge. Figures 7C,D showed the AUC_0-t_ values of schaftoside both on day 1 and day 6 were significantly sensitive to dissolution shape, kidney-volume, kidney-plasma clearance and fraction unbound. In addition, the AUC_0-t_ was also influenced by kidney-specific blood flow rate, intestinal permeability, large intestinal transit time and small intestinal transit time. However, the influences of gastric emptying time, solubility, pKa, and lipophilicity on AUC_0-t_ were not significant. Altogether, “dissolution shape”, “fraction unbound”, “kidney-volume”, and “kidney-plasma clearance” were identified to be the key factors that influenced the simulated schaftoside plasma concentration-time profiles in human.

### 3.6 PBPK simulation in elderly and renally impaired population

Following verification of the model in healthy volunteers, the PK-Sim was used to predict systemic exposure in elderly population and patients with “CKD *stage3*” (GFR = 60 ml/min/1.73 m^2^), “CKD *stage4*” (GFR = 30 ml/min/1.73 m^2^) and “CKD *stage5*” (GFR = 15 ml/min/1.73 m^2^). In order to further evaluate the safety of the drug in renally impaired patients, we also simulated healthy population taking a high dose of TFDS Capsules (1 g q 8 h), which was confirmed to be a safe dose in previous clinical study. The PK profiles of schaftoside in special subgroups were compared with those in the healthy population (18–40 years old).

**FIGURE 8:**
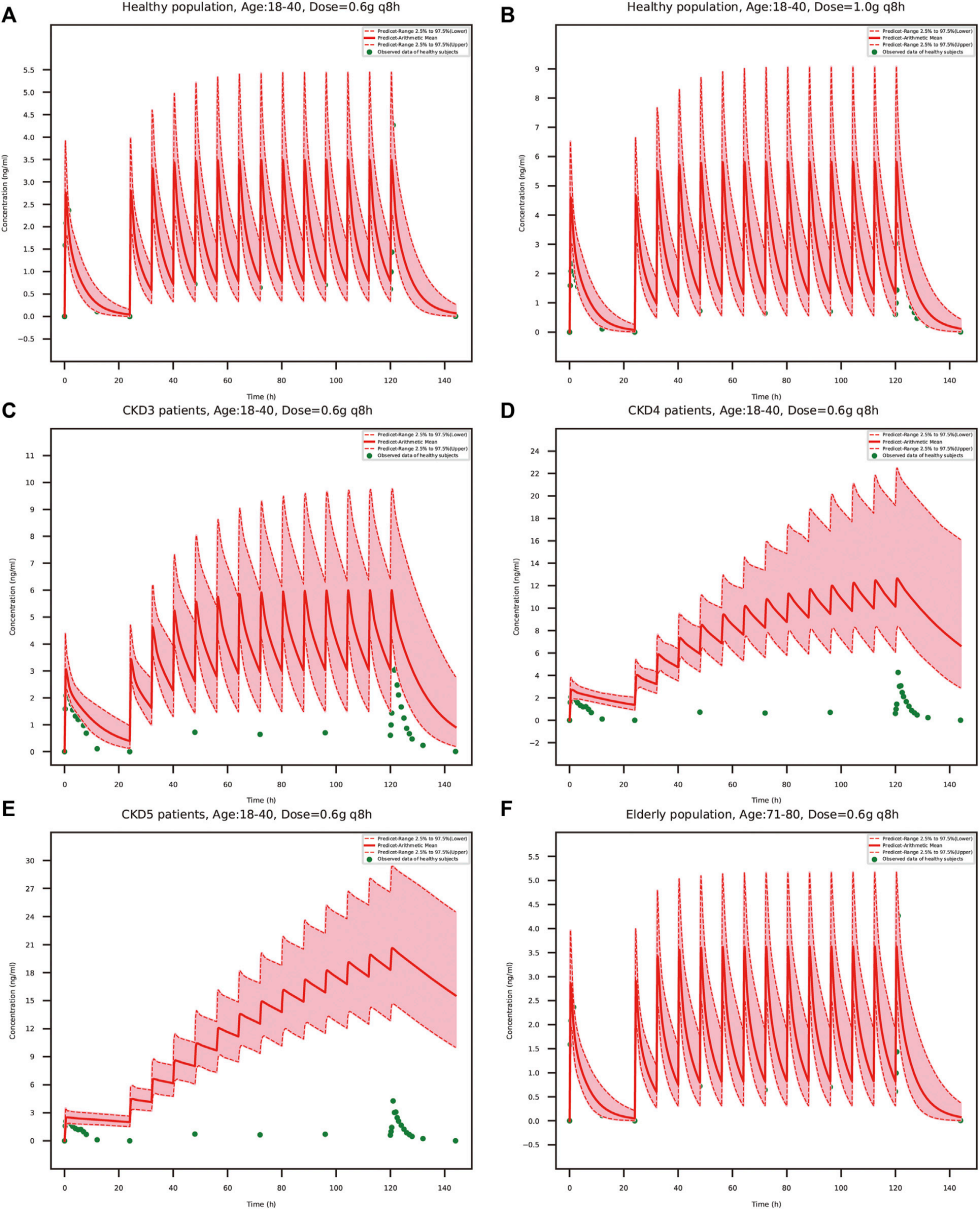
PBPK model-simulated schaftoside plasma concentration-time profiles in healthy population (18–40 years) at the TFDS regular dosage **(A)**: 0.6 g q 8 h) or a higher dosage **(B)**: 1 g q 8 h), renally impaired patients with CKD *stage3* **(C)**, GFR = 60 ml/min/1.73 m^2^), CKD *stage4* **(D)**, GFR = 30 ml/min/1.73 m^2^), CKD *stage5* **(E)**, GFR = 15 ml/min/1.73 m^2^), and elderly population **(F)**, 71–80 years). Simulated data included the mean values, 97.5th percentile and 2.5th percentile concentration range. The green solid circles represent mean observed data obtained from the healthy volunteers in the present clinical study.

The predicted schaftoside plasma concentration-time profiles for these populations (*n* = 100) are shown in Figure 8. The predicted C_max_ and AUC_0-t_ values of schaftoside in the high-dose group (1 g) were 1.67 times higher than that in the regular-dose group (0.6 g) following single- and multiple-dose administration of TFDS in healthy population. Compared to the healthy subjects, the plasma exposure of schaftoside was higher in CKD patients. After the single dose of 0.6 g TFDS, the mean AUC_0-t_ values in CKD3, CKD4, CKD5 patients were 2.14, 3.47, 4.02-fold of the data in healthy population (0.6 g), but the maximum plasma concentration of schaftoside did not differ significantly in these populations (Table 6). After multiple-dose administration (0.6 g q 8 h), the mean C_max_ and AUC_0-t_ values on day 6 were increased by 0.72, 2.62, 4.91-fold and 2.41, 11.32, 22.77-fold, respectively, in CKD3, CKD4, CKD5 patients. Additionally, compared to the high-dose group of TFDS (1 g) in the healthy population, the plasma exposure (C_max_ and AUC_0-t_) of schaftoside in the regular-dose group (0.6 g) were increased more than two times for the patients with CKD4-5. Furthermore, the drug exposure in the elderly population was explored in this study. The plasma concentrations of schaftoside after oral administration of TFDS did not vary greatly between elderly people (71–80 years) and adult population (18–40 years).

**TABLE 6:** Predicted pharmacokinetic parameters of schaftoside following oral administration of TFDS at 0.6 g/1 g q 8 h.

Parameter		Healthy (0.6 g q 8 h)		Healthy (1 g q 8 h)		CKD3 (0.6 g q 8 h)		CKD4 (0.6 g q 8 h)		CKD5 (0.6 g q 8 h)		Eldly (0.6 g q 8 h)	
		Day 1	Day 6	Day 1	Day 6	Day 1	Day 6	Day 1	Day 6	Day 1	Day 6	Day 1	Day 6
C_max_ (ng/mL)	Mean	2.76	3.49	4.6	5.82	3.06	6	2.72	12.65	2.52	20.64	2.86	3.62
	Range 2.5%	1.81	2.25	3.02	3.75	2.32	4.27	1.93	8.38	1.89	14.7	1.94	2.5
	Range 97.5%	3.91	5.44	6.52	9.07	4.39	9.76	3.82	22.49	3.49	29.38	3.96	5.17
R_Cmax_^a^	Mean	1.26		1.27		1.96		4.65		8.19		1.27	
	Range 2.5%	1.24		1.24		1.84		4.34		7.78		1.29	
	Range 97.5%	1.39		1.39		2.22		5.89		8.42		1.30	
AUC_0-t_	Mean	13.33	18.25	22.22	30.42	28.49	62.21	46.31	224.85	53.54	433.77	13.71	18.98
(h·ng/mL)	Range 2.5%	7.69	9.35	12.81	15.59	18.5	30.79	34.39	128.03	40.5	296.5	7.74	9.45
	Range 97.5%	22.05	34.91	36.75	58.19	44.21	133.06	60.96	451.02	69.65	647.54	23.02	39.48
R_AUC_^b^	Mean	1.37		1.37		2.18		4.86		8.10		1.38	
	Range 2.5%	1.22		1.22		1.66		3.72		7.32		1.22	
	Range 97.5%	1.58		1.58		3.01		7.40		9.30		1.72	

^a^ Ratio of C_max_ of schaftoside on day 6 *versus* day 1.

^b^ Ratio of AUC_0-t_ of schaftoside on day 6 *versus* day 1.

## 4 Discussion

With the rapid development of economies over the past few decades, the incidence of urolithiasis is increasing annually worldwide (Raheem et al., 2017). For example, the prevalence was increased from 5.95% to 10.63% during the period 1991–2016 in mainland China (Wang et al., 2017). Although surgery operations and various drugs are used to treat urolithiasis, high recurrence and side effects of drugs are still problematic. Flavonoids are a large group of plant polyphenols in nature which are endowed with pleiotropic activities, such as anti-crystallization of stones, antioxidant, anti-inflammatory and other protective effects (Zeng et al., 2019). Thus, flavonoids are presumed to have beneficial effects on the disease, yet a very few of them have reached clinical use. Therefore, it is exciting when the total flavonoids of *Desmodium styracifolium* (TFDS) have shown good efficacy and tolerability in randomized clinical trials. However, many unresolved questions remain, such as the PK profiles of active constituents in human, the safety and efficacy in patients with impaired kidney or liver function, and the dosage recommendations across a wide range of ages. To address these issues, we developed a PBPK model in human to predict the plasma concentration profile of schaftoside in different populations. This method could provide useful insight for safety evaluation and dose selection of TFDS.

There are many tricky problems to be solved about the development of PBPK models of herbal medicines. Herbal extracts usually contain complex chemical compositions, and interactions between the components may influence the establishment of PBPK models. Metabolism mediated by intestinal microflora or hepatic enzymes is one of the most important factors restricting the building of models (Meng and Liu, 2014; Feng et al., 2019). Therefore, we conducted *in vitro* and *in vivo* experiments to investigate the metabolic features of schaftoside. The *in vitro* results showed schaftoside underwent poor metabolism in rat and human liver microsomes, and *in vivo* studies demonstrated schaftoside was mainly excreted into the urine (∼55%) and bile (∼25%) in forms of the parent drug itself, rather than various metabolites. To compare the metabolic characteristics of schaftoside in pure form and mixture, we analyzed the drug components and metabolites in rats after oral administration of TFDS in previous studies, and no obvious phase I and II metabolites of schaftoside were observed (data not shown). These findings indicated the possibility of metabolism-mediated interactions was relatively small between schaftoside and other compounds in TFDS. In line with our results, Tremmel and colleagues reported schaftoside underwent poor metabolism in an intestinal and epithelial metabolism model (Tremmel et al., 2021). They found schaftoside, isoschaftoside, and vitexin were poorly metabolized in phase I and II reactions, while orientin, isoorientin, and isovitexin were highly metabolized. By comparing the structures of these C-glycosylated flavones, we speculated that the hydroxy groups at C-4’/C-5’ in the B-ring and sugar residues linked to C-8 in the A-ring were the meaningful structural features for the metabolism of these compounds.

TFDS contains several flavonoid glycosides including schaftoside, which are poorly soluble in nature. Therefore, the oral formulation for PK studies in rat is a TFDS suspension solution, which means the dissolution during administration may be incomplete. In order to simulate possible solubility, the dissolution time and dissolution shape in rat were set 0.92 min and 1.08*1E-3, respectively. Nevertheless, there was still an overestimation in schaftoside plasma concentration especially for C_max_. According to the literature, intestinal absorption may be limited by solubility, thus imposing an upper limit on the absorption of compounds (Gerner and Scherf-Clavel, 2021). Even so, the predicted C_max_ and AUC_0-t_ values of schaftoside after oral administration of TFDS in rats were within ∼1.5-fold of the observed values, which met the acceptance criteria (<2-fold). Likewise, the predicted profiles of schaftoside in human model were consistent with the observed data obtained from our clinical study, demonstrating the reliability and robustness of the PBPK model.

To evaluate the efficacy and safety of TFDS in various populations, the adult PBPK model in healthy population was scaled to elderly people and renally impaired patients. After multiple-dose administration, the predicted C_max_ and AUC_0-t_ of schaftoside on day 6 were obviously higher in CKD patients than the healthy people. The drug exposure increased with the decline of renal function. Compared to the healthy people, both the C_max_ and AUC_0-t_ values of schaftoside were increased more than two times in the CKD4-5 patients. We speculated that the renal function could greatly affect the excretion of schaftoside, and declined renal function in CKD patients led to the accumulation of shaftoside. The increased ratio of C_max_ and AUC_0-t_ of schaftoside on day 6 *versus* day 1 verified this speculation. Thus, exposure-related adverse events may be more frequently experienced by patients with impaired renal function during TFDS treatment. On the contrary, the drug exposure of schaftoside in the elderly population (71–80 years old) did not differ with the adult people (18–40 years old), indicating age was not a key factor influencing the safety and efficacy of TFDS.

There are some limitations to this study. One limitation which might reduce the applicability of the model was the lack of iv data of schaftoside in human (Gerner and Scherf-Clavel, 2021). Although the rat PBPK model utilized the iv and oral plasma concentration-time profiles of schaftoside and TFDS, the establishment of human PBPK model only based on the oral data of schaftoside after oral administration of TFDS. However, clearance processes could not be transferred from rat to human completely due to species differences. This may lead to some uncertainties and inaccuracies of PBPK models. However, the excellent model fit indexes such as FE, AFE and AAFE values which were within 2-fold error indicated the schaftoside PBPK model in human was generally accurate and reliable. We speculated it may be related to the metabolic characteristics of schaftoside in animals and human. Schaftoside underwent poor metabolism and was extensively excreted as an unchanged form. Another shortcoming of the present study was the relatively small clinical sample size (Gao et al., 2015) and the lack of pharmacokinetic data of TFDS in CKD patients. Because TFDS was recently approved for the treatment of urolithiasis, the clinical data was not adequate. Therefore, model validation in patients was not sufficient. In the future, more clinical data could be incorporated into the human PBPK model. Then the patient model can be refined and expanded with the availability of additional information. Third, the drug transporters were not included in the current PBPK models. It was reported drug transporters played important roles in the disposition of flavonoid C-glycosides. Schaftoside was identified as the substrate of multidrug resistance protein in previous studies (Fang et al., 2019; Song et al., 2020; Shen et al., 2021). A future investigation of the activities of drug transporters related with schaftoside will be proper for the optimization of PBPK models. Last, PBPK model was only applied to simulate pharmacokinetic profiles of schaftoside in this study. Although schaftoside is the representative compound of TFDS, we cannot exclude the important roles of the other constituents in TFDS such as vicenin-1, vicenin-2, and vicenin-3. More studies need to be performed to investigate their pharmacology activities and pharmacokinetic profiles in the future.

## 5 Conclusion

In conclusion, the established PBPK models of schaftoside in rats and humans are both reliable and valid. Using this model, we successfully simulated the pharmacokinetic profiles of schaftoside in elderly and renally impaired patients after oral administration of TFDS. The results indicated there is no PK-based need for dose adjustment in elderly population, however, dosage reduction may be recommended for patients with severe renal impairment. This study provided a feasible way for the assessment of efficacy and safety of herbal medicines.

## Data Availability

The raw data supporting the conclusion of this article will be made available by the authors, without undue reservation.
